# PCR-Based Identification of *Klebsiella pneumoniae* subsp. *rhinoscleromatis*, the Agent of Rhinoscleroma

**DOI:** 10.1371/journal.pntd.0001052

**Published:** 2011-05-24

**Authors:** Cindy Fevre, Virginie Passet, Alexis Deletoile, Valérie Barbe, Lionel Frangeul, Ana S. Almeida, Philippe Sansonetti, Régis Tournebize, Sylvain Brisse

**Affiliations:** 1 Institut Pasteur, Genotyping of Pathogens and Public Health, Paris, France; 2 CEA-IG, Genoscope, Evry, France; 3 Institut Pasteur, Intégration et Analyse Génomique, Paris, France; 4 Institut Pasteur, Unité de Pathogénie Microbienne Moléculaire, Paris, France; 5 Unité INSERM U786, Institut Pasteur, Paris, France; Yale University School of Medicine, United States of America

## Abstract

Rhinoscleroma is a chronic granulomatous infection of the upper airways caused by the bacterium *Klebsiella pneumoniae* subsp. *rhinoscleromatis*. The disease is endemic in tropical and subtropical areas, but its diagnosis remains difficult. As a consequence, and despite available antibiotherapy, some patients evolve advanced stages that can lead to disfiguration, severe respiratory impairment and death by anoxia. Because identification of the etiologic agent is crucial for the definitive diagnosis of the disease, the aim of this study was to develop two simple PCR assays. We took advantage of the fact that all *Klebsiella pneumoniae* subsp. *rhinoscleromatis* isolates are (i) of capsular serotype K3; and (ii) belong to a single clone with diagnostic single nucleotide polymorphisms (SNP). The complete sequence of the genomic region comprising the capsular polysaccharide synthesis (*cps*) gene cluster was determined. Putative functions of the 21 genes identified were consistent with the structure of the K3 antigen. The K3-specific sequence of gene Kr11509 (*wzy*) was exploited to set up a PCR test, which was positive for 40 K3 strains but negative when assayed on the 76 other *Klebsiella* capsular types. Further, to discriminate *Klebsiella pneumoniae* subsp. *rhinoscleromatis* from other K3 *Klebsiella* strains, a specific PCR assay was developed based on diagnostic SNPs in the phosphate porin gene *phoE*. This work provides rapid and simple molecular tools to confirm the diagnostic of rhinoscleroma, which should improve patient care as well as knowledge on the prevalence and epidemiology of rhinoscleroma.

## Author Summary

In humans, the bacterium *Klebsiella pneumoniae* subsp. *rhinoscleromatis* (also called clone Rhinoscleromatis, as it evolved from a single *Klebsiella pneumoniae* ancestral strain) causes rhinoscleroma, a chronic infection of the nose and throat. Identification of the bacterium from biopsies or nasal secretions is essential for diagnosis, and currently relies on the biochemical characteristics of clone Rhinoscleromatis and on detection of its capsule of antigenic type K3. Our aim was to develop two identification tests based on amplification by polymerase chain reaction (PCR) of specific portions of the genome of clone Rhinoscleromatis. We established the sequence of the capsular polysaccharide synthesis cluster and identified one gene sequence that was unique to K3 strains. A PCR test that targets this gene was shown to be specific for K3 strains. We also exploited unique DNA signatures of clone Rhinoscleromatis to develop a second PCR test, which is specific for this clone, thus allowing distinction from other *K. pneumoniae* K3 strains. These novel and simple identification tests should help to diagnose rhinoscleroma and to understand the epidemiology of this disease.

## Introduction

Rhinoscleroma is a chronic granulomatous infection of the nose and upper airways of humans. The relative rarity of the disease and low specificity of early symptoms make the clinical diagnosis of rhinoscleroma difficult [1]. As a consequence, some patients evolve advanced stages that can lead to severe respiratory impairment [2]. The most characteristic feature of the disease is the observation in biopsies of big foamy cells, called Mikulicz cells, in which bacilli are seen after hematoxilin-eosin staining [3]. Hence, the specific diagnosis of rhinoscleroma currently relies on the combination of histological and bacteriological examinations, with the presence of Mikulicz cells and the identification of the causative agent, the bacterium *Klebsiella pneumoniae* subsp. *rhinoscleromatis* [4] from tissue culture, nasal swabs or blood culture [4], [5].

Initially, species status (*Klebsiella rhinoscleromatis*) was given to the agent of rhinoscleroma. However, given high similarity based on DNA-DNA hybridization [6], it is now regarded as a subspecies of *Klebsiella pneumoniae* [7]. Multiple gene sequencing confirmed the close relatedness of the agent of rhinoscleroma with *K. pneumoniae* [8]–[10]. Currently, the definitive identification of the organism is difficult. Different from *K. pneumoniae* sensu stricto (i.e., *K. pneumoniae* subsp. *pneumoniae*), which can be encountered in many hosts, environments and clinical sources [11], *K. pneumoniae* subsp. *rhinoscleromatis* is strictly associated with humans and has, to our knowledge, only been isolated from cases of rhinoscleroma. As *K. pneumoniae* subsp. *rhinoscleromatis* is not considered as a commensal of the respiratory tract, its identification is key to the definitive diagnosis of the disease [12], [13]. This infectious agent is metabolically less versatile than other *K. pneumoniae* strains, but definitive identification based on biochemical properties (ONPG negative, no acid production from lactose, urease, LDC and citrate negative) is rendered difficult due to variation in metabolic characteristics of *K. pneumoniae* strains [9], [11]. Furthermore, members of a third subspecies, *K. pneumoniae* subsp. *ozaenae*, which are associated with an atrophic rhinitis known as ozaena, also have reduced metabolic capacities [8].

*Klebsiella* strains are typically surrounded by a polysaccharidic capsule of considerable thickness that is responsible for the mucoid aspect of colonies. *Klebsiella* capsular serotyping (K typing) distinguishes at least 77 K types [7], [14]. As all strains of *K. pneumoniae* subsp. *rhinoscleromatis* are of capsular type 3 (K3), K typing is used for confirmatory identification of *K. pneumoniae* subsp. *rhinoscleromatis* and allows distinction from *K. pneumoniae* subsp. *ozaenae* strains, which are mostly of type K4 and more rarely of types K5 and K6 [7]. However, K typing is technically cumbersome and a small proportion of *K. pneumoniae* subsp. *pneumoniae* strains (including the type strain CIP 82.91^T^ = ATCC 13883^T^) are of type K3 as well, meaning that capsular typing alone cannot provide a definitive identification of *K. pneumoniae* subsp. *rhinoscleromatis*. Restriction fragment length polymorphism (RFLP) of the amplified capsular polysaccharide synthesis (*cps*) region [15] distinguished, among strains of the K3 capsular type, four distinct banding patterns (C-patterns C3a to C3d) that differ by one or a few DNA fragments [8]. All *K. pneumoniae* subsp. *rhinoscleromatis* isolates have C-pattern C3a, but some *K. pneumoniae* subsp. *pneumoniae* isolates also have this pattern, thus impairing the use of this method to confirm identification of the agent of rhinoscleroma.

Our recent population genetics study [8] showed that isolates of *K. pneumoniae* subsp. *rhinoscleromatis* are genetically homogeneous and form a unique clonal family (clonal complex CC67) that can be distinguished, based on the sequence of housekeeping genes, from all other *K. pneumoniae* members, including *K. pneumoniae* subsp. *ozaenae*. Hence, these isolates can be regarded as members of a clone that evolved recently from its ancestor, and we hereafter use the designation clone Rhinoscleromatis for this recent phylogenetic entity, as this clone comprises all studied isolates that belong to *K. pneumoniae* subsp. *rhinoscleromatis*, and only these isolates. The loss of metabolic abilities by clone Rhinoscleromatis was proposed to reflect its ecological specialization to the human respiratory tract [8]. Based on multilocus sequence typing (MLST), *K. pneumoniae* subsp. *pneumoniae* strains of the capsular type K3 are genetically heterogeneous but, importantly, all are clearly distinct from clone Rhinoscleromatis. Indeed, the presence of capsular type K3 in different genetic backgrounds can be explained by past events of horizontal transfer of the *cps* region [8]. Interestingly, two synonymous SNPs located at positions 48 and 216 on the phosphoprotein gene *phoE*, were observed in all isolates of clone Rhinoscleromatis, but in no other *K. pneumoniae* strain. Phosphoporin E is an outer membrane pore protein with a recognition site for negatively charged compounds, the expression of which is induced under phosphate limitation.

The aims of this study were (1) To characterize the structure and gene content of the *Klebsiella* K3 *cps* cluster and to use its unique features to set up a specific PCR assay for K3 strains; and (2) To develop a specific molecular assay to identify members of clone Rhinoscleromatis and distinguish them from other *K. pneumoniae* strains, including those of capsular type K3.

## Methods

### Identification and sequencing of strain SB3432

The clone Rhinoscleromatis strain SB3432 was isolated in 2004 in the Avicenne hospital, Bobigny, France from a biopsy of the left nasal cavity of an 11-year old patient diagnosed with rhinoscleroma. Identification was initially achieved using API NE and API20E strips (BioMerieux, Marcy-l'Etoile, France), and by biochemical characteristics [11]. MLST [16] confirmed this strain to be 100% identical at seven housekeeping genes to the type strain of *K. pneumoniae* subsp. *rhinoscleromatis* CIP 52.210^T^ ( = ATCC 13884^T^). The *cps* PCR-RFLP pattern was determined as C3a by the molecular serotyping method [15]. In the frame of the complete genome sequencing of Rhinoscleromatis strain SB3432, the *cps* cluster sequence was determined. The K3 *cps* cluster sequence was deposited in GenBank/EMBL/DDBJ databases under accession number FQ311478.

### Annotation of the K3 *cps* cluster

Open reading frames (ORFs) encoding proteins of ≥40 amino acids were evaluated for coding potential using the Genemark program [17]. Other criteria included similarity to other *Klebsiella* and gene overlaps were used to propose a start codon. Homology searches were also conducted using TBLASTX [18] on Uniprot [19] and COG dababases [20]. Finally, each putative CDS was curated manually through visual inspection within the CAAT-Box programme [21]. Topology prediction of membrane proteins was achieved with the TopPred program [22].

### Comparison of *cps* clusters

**Figure 1:**
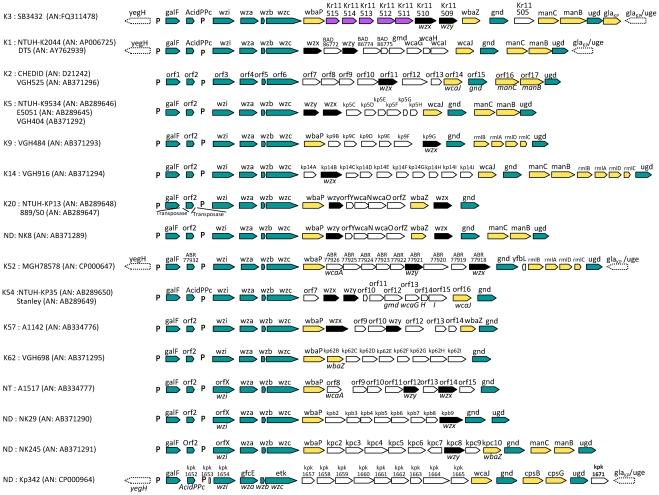
Gene composition of the *cps* region of *Klebsiella* capsular serotype K3 and comparison with other *cps* regions. The comparative strains represent 11 serotypes (K1, K2, K3, K5, K9, K14, K20, K52, K54, K57 and K62), one new serotype (NT) and four undetermined serotypes (ND). Arrows with dotted lines belong to flanking regions of the *cps* cluster. Green arrows represent highly conserved genes present in all *cps* regions. Yellow arrows correspond to genes of the K3 *cps* with highly conserved sequences, but which presence/absence depends on capsular type. Serotype K3 genes having weaker or no homology with the other *Klebsiella cps* regions are represented by purple arrows. Black arrows correspond to genes *wzx* and *wzy*, which are highly variable at the sequence level. In the K3 *cps* region, the suggested names for the ORFs in green and yellow are based on amino-acid homology superior to 78%, with at least one annotated gene from another *Klebsiella cps* regions. For the previously published *cps* regions, names indicated above each gene correspond to annotations found in public databases, whereas names in italic under the genes are suggestions based on our pairwise comparisons (>65% amino acid identity except for *wzx* and *wzy*). The suggested *wzx* and *wzy* annotations are based on 30% to 48% and 23% to 26% of amino-acid similarity on most of their sequence length with the respective proteins of *E. coli*. As noted earlier [28], the presence of genes *wbaP* and *wcaJ* appeared mutually exclusive. P: promoter [27]; AN: Accession number.

In order to compare the *cps* region encoding a capsular type K3 with previously sequenced *cps* regions of *K. pneumoniae*, all genes from *galF* (UTP-glucose-1-phosphate uridylyltransferase) to *gla_KP_* (homonym of *uge* [23], [24], encoding an UDP-galacturonic acid C4-epimerase) were submitted to pairwise comparison using clustalW2 algorithm (http://www.ebi.ac.uk/Tools/clustalw2/index.html). Previously published *cps* regions of *Klebsiella* strains were used: serotype K1 (strains NTUH-K2044 [25] and DTS [26]), K2 (strains CHEDID [27] and VGH525 [28]), K5 (strains NTUH-K9534, E5051 [29] and VGH404 [28]), K9 (strain VGH484 [28]), K14 (strain VGH916 [28]), K20 (strains NTUH-KP13 and 889/50 [29]), K52 (strain MGH78578), K54 (strain NTUH-KP35 [29]), K57 (strain A1142 [30]), K62 (strain VGH698 [28]) as well as a non-typeable strain (A1517 [30]) and four strains with undetermined serotype (NK8, NK29, NK245 [28] and Kp342 [31]). Accession numbers are given on **Figure 1**.

### PCR assay for K3 *Klebsiella* strains

Primers Kr11509-F (5′- TAG GCA ATT GAC TTT AGG TG - 3′) and Kr11509-R (5′- AGT GAA TCA GCC TTC ACC T - 3′) were designed inside the sequence of ORF Kr11509 (*wzy*) to allow PCR amplification of a 549 bp fragment. The PCR mix contained 0.85 U of Taq polymerase (Invitrogen), 100 µM of each deoxynucleoside triphosphate, 0.2 µM of each primer, 40 ng of DNA, 1.5 mM MgCl_2_, 20 mM Tris-Hcl (pH 8.4) and 50 mM KCl in a final volume of 50 µl. Samples were submitted to an initial denaturation step (30 sec at 94°C), followed by 35 amplification cycles (30 sec at 94°C, 30 sec at 52°C, 30 sec at 72°C) and a final elongation step (5 min at 72°C). A MasterCycler ep Gradient S thermocycler (Eppendorf) was used. This K3 PCR assay was tested on 164 *Klebsiella* strains from a previous study [8], including 16 clone Rhinoscleromatis isolates and 14 K3 *K. pneumoniae* subsp. *pneumoniae* strains (**Table S1**). As a control that DNA was PCR-amplifiable, *rpoB* (RNA polymerase beta-subunit) PCR was performed as described previously [16]. Water was used as template for negative controls.

### *phoE* PCR assay for clone Rhinocleromatis

Primer pair phoE-rhiF (5′-GCG GCA GCG ACT TCG CCG TA-3′) and phoE-rhiR (5′-GTT CTG CGC TTT GTT GGC AAA C-3′) were designed so that their 3′ base corresponded to the two Rhinoscleromatis-specific SNP positions. PCR mix composition and the thermocycler used were identical to those used for K3 PCR. Samples were submitted to an initial denaturation step (30 sec at 94°C), followed by 35 amplification cycles (30 sec at 94°C, 30 sec at 58°C, 30 sec at 72°C) and a final elongation step (5 min at 72°C). This PCR assay was tested on 42 *Klebsiella* strains, including 16 clone Rhinoscleromatis isolates, 20 *K. pneumoniae* subsp. *pneumoniae* strains, five *K. pneumoniae* subsp. *ozaenae* strains and one *K. planticola* (**Table S1**). *rpoB* PCR was used as positive control, while water was used for negative controls.

### Animal infections

Female Balb/cJ mice were used at the age of 6–8 weeks and housed in dedicated biosafety level 2 animal facilities of Institut Pasteur. Mice received food and water ad libidum. All animal experiments were carried according to the French national guidelines for animal experiments and were approved by the Animal Care Committee of the Institut Pasteur with the agreement number 05-59. Mice were infected by intra-nasal inoculation of 2.10^6^ or 2.10^7^ *K. pneumoniae* subsp. *rhinoscleromatis* strain SB3432. The control group was inoculated with the same volume of saline water. Five days after infection, lungs were surgically removed for bacterial load quantification and total DNA extraction. Briefly, lungs were collected and homogenized mechanically in 3 ml of ice-cold Hepes buffer supplemented with 0.2 mM EDTA and 0.1% bovine serum albumin. Bacterial counts were determined as colony forming units (CFU) by plating serial dilutions of the lung extract. Total DNA was extracted using a Genomic DNA Isolation NucleoBond kit (Macherey-Nagel), following the manufacturer's instructions.

## Results

### 1. K3 *cps* operon structure and identification of a K3-specific gene sequence

We determined the complete sequence of the *cps* region of the capsular type K3 *K. pneumoniae* subsp. *rhinoscleromatis* strain SB3432. The 21 ORFs that are likely to represent coding sequences in the region between *galF* and *gla_KP_* were compared with the *cps* region of the other *Klebsiella* capsular types sequenced to date (**Table 1**, **Figure 1**) [25]–[31]. Eight genes (*galF*, acid phosphatase gene, *wzi*, *wza*, *wzb*, *wzc*, *gnd* and *ugd*; green arrows in **Figure 1**) were highly conserved, with homologs in all *Klebsiella cps* regions and in the group 1 capsule locus of *E. coli* [32]. The JUMPstart (for “just upstream of many polysaccharide starts”) element, involved in transcriptional antitermination of the *cps* cluster [33], is present upstream of gene *wzi*. In all strains for which the sequence downstream of *gnd* was available, gene *ugd* was found and considered to be the last gene of the *cps* cluster [28], [29]. In the three fully sequenced strains NTUH-K2044, MGH78578 and Kp342, *ugd* is followed by gene *gla_KP_* (**Figure 1**), also known as *uge* [23] and involved in galacturonic acid synthesis [24]. This gene is in the opposite direction and considered to be part of the cluster for synthesis of the LPS core [24], [34], [35]. In the K3 *cps* cluster, this *gla_KP_* gene is preceded by another, face-to-face copy of *gla_KP_* (**Figure 1**). Because the K3 capsular polysaccharide contains a galacturonic acid, which is rare among known *Klebsiella* polysaccharide structures [7], the forward *gla_KP_* copy might represent the last gene of the K3 *cps* cluster.

**Table 1:** Composition of the capsular type K3 *cps* cluster.

Putative function	Gene	Gene name/annotation	Best BLAST hit	Amino-acid identity	Species (serotype/strain name) 1
Polymerization and surface assembly	*wzi*	Outer membrane protein	*wzi*	99%	Kp (K20, K52, K57, NK8, NK29, NK245)
Polymerization and surface assembly	*wza*	Multimeric putative translocation channel	*wza*	92%	Kp (all serotypes except K2 and NK8)
Polymerization and surface assembly	*wzb*	Protein tyrosine phosphatase	*wzb*	78%	*E. coli* (K30), Kp (K20, NK8)
Polymerization and surface assembly	*wzc*	Inner membrane tyrosine autokinase	*wzc*	81%	*E. coli* (K30), Kp (K20, NK8)
Polymerization and surface assembly	Kr11510	Putative flippase *wzx*	*wzx*	25%	*Streptococcus pneumoniae* (23% on *E. coli* wzx; 24% on K57 wzx)
Polymerization and surface assembly	Kr11509	Putative polymerase *wzy*	*wzy*	15%	Kp (K20, K54, K57)
D-galactose synthesis	*galF*	UTP-glucose-1-phosphate uridylyltransferase	*galF*	100%	Kp (K1, K52)
Unknown	*AcidPPC*	acid phosphatase homolog	*AcidPPc*	100%	Kp (K14)
Unknown	*gnd*	gluconate-6-phosphate dehydrogenase	*gnd*	99%	Kp (K14, K54)
Synthesis of UDP-glucuronic acid from UDP-glucose	*ugd*	UDP-glucose 6-dehydrogenase	*ugd*	100%	Kp (K9, NK245, Kp342), *E. coli*
Transfer of D-galactose on undecaprenyl-phosphate	*wbaP*	Gal::undecaprenolphosphate Gal-1-P transferase	*wbaP*	84%	*E. coli* K30, Kp (K20, NK8)
Transfer of the first mannose residue on the galactose residue	*wbaZ*	Mannosyl transferase	*wbaZ*	76%	Kp (K57)
Synthesis of GDP-mannose from mannose-1-phosphate	*manC*	GDP-mannose phosphorylase	*manC/cpsB*	99%	Kp (K2, K14, NK245, Kp342)
Synthesis of mannose-1-phosphate from mannose-6-phosphate	*manB*	Phosphomannomutase	*manB/cpsG*	99%	Kp (K1, K2, K5, K14, K62, NK245, NK8, Kp342)
Synthesis of UDP-galacturonic acid from UDP-glucuronic acid	*glaKp/uge*	UDP-galacturonic C4-epimerase	*uge*	86%	Kp (Kp342)
Transfer of the second and third mannose residue	Kr11515	Putative mannosyl transferase	Mannosyl transferase	47%	*Salmonella enterica* subsp. *enterica*
Unknown	Kr11511	Putative mannosyl transferase	Mannosyl transferase	39%	*Bacteroides fragilis*
Transfer of the UDP-galacturonic acid on the second mannose residue	Kr11513	Putative group 1 glycosyl transferase	Group 1 glysosyl transferase	55%	*Serratia proteamaculans*
Unknown	Kr11514 Kr11512	Hypothetical protein	Hypothetical protein	47%	*Serratia proteamaculans*
Transposition of mobile genetic element	Kr11505	Transposase	Transposase	98%	*E. coli*

1 Kp, *Klebsiella pneumoniae*.

Four other genes of the K3 *cps* region (*wbaP*, *wbaZ*, *manB* and *manC*; **Figure 1**, yellow arrows) were highly similar to genes in other *cps* clusters (**Table 1**), but their presence depended on K type (**Figure 1**). Further, five genes of the K3 *cps* cluster (Kr11511 to Kr11515; **Figure 1**, purple arrows) had weaker but nevertheless significant homology (**Table 1**) with genes previously described in *Klebsiella cps* clusters, or with genes in other species. The K3 *cps* cluster also encodes a putative transposase (Kr11505), located between *gnd* and *manC* but not found in the other *cps* regions.

Finally, the sequence of both *wzx* (flippase) and *wzy* (polymerase) genes, necessary for polysaccharide synthesis in *E. coli*, is known to be highly variable among capsular types, rendering their identification problematic [28], [34]. The ∼25% identity with Wzx in different species might indicate that ORF Kr11510 corresponds to the flippase gene (**Table 1**). Likewise, ORF Kr11509 showed 9.9% to 15.8% similarity with Wzy proteins in other *Klebsiella cps* clusters, which is the range of the similarity among other *Klebsiella* Wzy proteins (9.2% to 19%). Moreover, topology prediction revealed that Kr11509 encodes a protein with eleven transmembrane domains, which is typical for Wzy sugar polymerases. As no other gene in the *cps* operon or elsewhere in the genome presented homology with *wzy*, we hypothesize that ORF Kr11509 encodes the K3 capsule polymerase.

### 2. Development of a PCR assay specific for K3 *Klebsiella* strains

**Figure 2:**
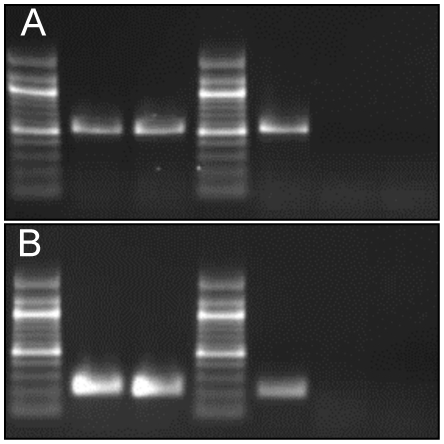
K3 and *phoE* PCR assays. (A) Kr11509 (*wzy*) PCR amplification, which is specific for serotype K3 *Klebsiella* isolates. Serotype K3 isolates of *K. pneumoniae* subsp. *pneumoniae* (lane 2: strain SB3204 = CIP 52.146; lane 3: strain SB3206 = CIP 82.91^T^) and subsp. *rhinoscleromatis* (lane 5: strain SB3432) showed the expected PCR product of 549 bp. This result is representative of the 16 clone Rhinoscleromatis isolates and the 14 K3 *K. pneumoniae* subsp. *pneumoniae* strains tested in this study (Table S1). No PCR amplification was obtained with the 134 non-K3 *Klebsiella* isolates tested (Table S1) representing the 76 other serotypes, as shown for serotype K2 (lane 6: strain SB3341 = CIP 52.145) and serotype K4 (lane 7: strain SB3220 = *K. pneumoniae* subsp. *ozaenae* CIP 52.211^T^). Lanes 1 and 4: 100 bp ladder (New England Biolabs). (B) *phoE* PCR amplification, which is specific for members of clone Rhinoscleromatis. PCR performed with strains belonging to clone Rhinoscleromatis (lane 2: strain SB167 = C5046; lane 3: strain SB1782 = *K. pneumoniae* subsp. *rhinoscleromatis* CIP 52.210^T^ and lane 5: strain SB3432) show the expected PCR amplification product of 209 bp. This product was observed for the 16 clone Rhinoscleromatis isolates tested in this study. PCR with non-Rhinoscleromatis *Klebsiella* strains (Table S1), including strains of serotype K3 and belonging to *K. pneumoniae* subsp. *ozaenae* gave no amplification (lane 6: SB3206 = CIP 82.91^T^; lane 7: *K. pneumoniae* subsp. *ozaenae* SB3220 = CIP 52.211^T^). Lanes 1 and 4: 100-bp ladder (New England Biolabs).

In order to develop a K3-specific PCR test, a pair of PCR primers (Kr11509-F and Kr11509-R) was designed to amplify a region of ORF Kr11509 (*wzy*). The PCR was assayed on 16 isolates of clone Rhinoscleromatis (all being K3), 14 K3 *K. pneumoniae* subsp. *pneumoniae* strains of C-patterns C3a to C3d, and 134 *Klebsiella* strains that included reference strains of the 76 other K types (**Table S1**). PCR amplification was positive in all K3 strains (30/30, 100%), and negative for all other strains (**Table S1** and **Figure 2A**). The *rpoB* PCR, used as positive control, was positive in all strains, while water controls were PCR negative. Therefore, this PCR assay was specific for K3 strains.

### 3. Development of a PCR assay specific for clone Rhinoscleromatis

To develop a PCR assay that distinguishes clone Rhinoscleromatis from all other *K. pneumoniae* clones, MLST data [8] were analyzed in order to find diagnostic single nucleotide polymorphisms (SNP) for clone Rhinoscleromatis. Two SNPs were identified on the phosphoporin gene *phoE*: the cytosines in position 48 and 216 (relative to position 1 of the MLST template, see www.pasteur.fr/mlst) observed in all *K. pneumoniae* subspecies *pneumoniae* and *K. pneumoniae* subspecies *ozaenae* strains, were replaced by an adenine and a guanine, respectively, in clone Rhinoscleromatis. The two SNPs are synonymous and correspond to positions 609 and 777 of the phosphoprotein *phoE* gene on the complete genome sequence of strain NTUH-K2044 (Accession number AP006725.1). We sought to exploit these two diagnostic SNPs located 168 bp apart on gene *phoE*, by developing an allele-specific PCR assay. The resulting *phoE* PCR was assayed on the 16 clone Rhinoscleromatis isolates, on 14 *K. pneumoniae* subsp. *pneumoniae* K3 strains, as well as on five *K. pneumoniae* subsp *ozaenae* strains, on six *K. pneumoniae* subsp. *pneumoniae* K1 and K2 isolates and on one *K. planticola* (**Table S1**). The expected PCR product was observed only after amplification of DNA from strains that were determined to belong to clone Rhinoscleromatis based on MLST, while all other strains were negative (**Table S1** and **Figure 2B**). The *rpoB* control PCR was positive in all strains and water controls were PCR negative. Hence, the *phoE* PCR assay was specific for clone Rhinoscleromatis, and positive for all strains of this clone.

### 4. Specificity, sensitivity and direct detection from biological samples

To investigate the potential use of the two PCR assays for direct diagnosis of rhinoscleroma, we first checked their specificity towards different pathogens. Both K3 and *phoE* PCR assays were negative at various dilutions of total DNA extracts from the bacterial throat commensals and pathogens *Neisseria meningitidis*, *Streptococcus pneumoniae*, *Streptococcus mitis*, *Streptococcus salivarius*, *Streptococcus mutans* and *Staphylococcus epidermidis*, whereas control 16S rRNA gene PCR amplifications of these extracts were positive (not shown). The sensitivity of the two PCR assays was tested by serial dilutions. The lowest quantity of total DNA extract yielding a clearly visible PCR product on ethidium bromide-stained agarose gels was 0.1 ng (*phoE* PCR) or 1 ng (K3 PCR), corresponding to 2.10^5^ and 2.10^6^ bacterial chromosomes, respectively. We then controlled and observed that human DNA alone did not yield any PCR product, even with large template amounts. We also demonstrated the robustness of the PCR assays to the presence of human DNA, as amplification from the clone Rhinoscleromatis DNA was still positive in the presence of up to 10-fold (K3 PCR) and 1000-fold (*phoE* PCR) excess of human DNA. Finally, we tested direct detection of the rhinoscleroma bacillus from lungs of experimentally infected mice (Fevre et al., in preparation). Five days post-infection with 2.10^6^ or 2.10^7^ bacilli, the number of the infecting agents was quantified from lung extracts. There were 4.10^7^ and 8.10^9^ CFUs per lung, respectively. In the same samples, the two PCR assays were strongly positive from total lung DNA of infected mice at DNA amounts of 0.1 ng (*phoE* PCR) and 1 ng (K3 PCR) and above. As the total DNA amount of lungs carrying 8.10^9^ cfu is 1.5 mg, this indicates that we can detect in vivo around 5.10^3^ bacteria per lung.

## Discussion

Rhinoscleroma remains difficult to diagnose [5], [36], [37]. The clinical symptoms depend on the region of the respiratory tract that is infected, and are often non-specific, mimicking those of other nasal disorders [37], [38]. Failure to diagnose rhinoscleroma is unfortunate, as treatments consisting in prolonged antibiotherapy with quinolones [39] or trimetoprim-sulfometoxazole [5] are available. Identification of the causative organism provides definitive diagnostic, but culture from biopsy can fail in a substantial number of cases [4], [5]. To our knowledge, there is no available molecular detection test. As all isolates of clone Rhinoscleromatis are of serotype K3, we followed the ‘molecular serotyping’ strategy, whereby the serotype is deduced from the sequence characteristics of the *cps* cluster, *e.g.* PCR-based identification of K types [30], [40], [41]. For this purpose, we established the complete sequence of the *cps* cluster of a K3 Rhinoscleromatis strain. We then developed two specific PCR assays for the identification of the agent of rhinoscleroma.

The deduced gene content of the K3 *cps* cluster is highly consistent with the structure of the K3 capsular polysaccharide from *Klebsiella* [42], as depicted on **Figure S1**. Currently, the link between capsular type 3 and *Klebsiella* strain virulence remains to be established. As the capsule is a prominent factor in bacterial pathogenesis, the availability of the K3 *cps* sequence will facilitate research into the possible implication of the K3 capsule in the peculiar pathophysiological aspects of rhinoscleroma.

Given the high sequence variability of *wzy* among distinct K-types, this gene is a good target for K-type specific PCR assays [28], [41]. The positive PCR results obtained herein for all K3 strains, belonging either to *K. pneumoniae* subsp. *pneumoniae* or to clone Rhinoscleromatis, indicate that *wzy* is conserved among K3 isolates, consistent with the fact that the *cps* PCR-RFLP patterns of K3 strains are highly similar [15]. This PCR assay can therefore be used to identify strains with capsular type 3, which would be technically easier than performing *Klebsiella* K typing with antisera. However, even though they are rare, some *K. pneumoniae* subsp. *pneumoniae* isolates also express the K3 capsular type [8], hampering the use of this PCR assay alone to identify the agent of rhinoscleroma. In contrast, MLST circumscribes clone Rhinoscleromatis without ambiguity [8] and as the phosphoporin gene *phoE* harbored diagnostic SNPs of this clone, we were able to develop a PCR assay that showed complete specificity with no false negatives. This achievement confirms that MLST data can be used to set up simple and rapid assays for identification of particular clones [43]. The two nucleotide changes at *phoE* positions 48 and 216 evolved in the branch leading to clone Rhinoscleromatis from its ancestor *K. pneumoniae*, since these changes were not observed in >700 other *Klebsiella* (including *K. granulomatis*), *Enterobacter* or *Raoultella* strains (our unpublished data and http://www.pasteur.fr/mlst). As the two mutations do not induce amino-acid changes, they can be considered neutral, and consequently, evolutionarily stable. For the same reason, it would be very unlikely that both mutations would evolve separately in other species.

The PCR assays were positive even in the presence of an excess of human DNA, and we were able to detect the infecting agent from the lungs of experimentally infected mice. In contrast, the assays were negative when tested on other pathogens and commensal organisms of the throat. Based on these preliminary observations, it should be interesting to evaluate the clinical value of these assays for direct detection of the agent of rhinoscleroma from human biopsies or nasal secretions.

In summary, although the *phoE* assay is, on its own, specific for clone Rhinoscleromatis, the combination of *phoE* and K3 PCR assays is recommended to increase the level of confidence in identifying clone Rhinoscleromatis. When combined with the defining metabolic characteristics of *K. pneumoniae* subsp. *rhinoscleromatis* [11], these two new PCR assays will be useful in confirming the diagnostic of rhinoscleroma and will help defining the epidemiology of this neglected disease.

## Supporting Information

Figure S1 Model of the biosynthesis, polymerization and surface assembly of the K3 capsular polysaccharide. A. Structure of the K3 polysaccharide [42]. B. Possible implication of genes of the cps region in the synthesis and expression of the K3 capsular polysaccharide. In Klebsiella and E. coli [28], [34], the synthesis of UDP-D-galactose from UDP-D-glucose requires protein GalE (UDP-galactose 4-epimerase), with GalF regulating its level [28]. Next, WbaP is involved in the transfer of the D-galactose to undecaprenyl-phosphate, the lipid carrier of repeat units [44]. Synthesis of D-mannose from mannose-6-phosphate is catalyzed by the products of genes manB and manC [44], which are located at the 3′ terminus of the K3 cps region. The (1→3) linkage of the D-mannose to D-galactose is performed by the mannosyl transferase encoded by wbaZ [34]. The linkage of the two additional D-mannose residues is presumably carried out by the putative mannosyl transferases Kr11515 and Kr11511. The remaining putative transferase of the K3 cps cluster with no assigned function, encoded by Kr11513, might be involved in the transfer of UDP-D-galacturonic acid residues on D-mannose. Synthesis of D-galacturonic acid residues is achieved by both Ugd and GlaKP enzymes. The product of gene ugd transforms UDP-D-glucose into UDP-D-glucuronic acid, which can be converted into UDP-D-galacturonic acid through the activity of GlaKP [45]. These capsule unit repeats are then translocated through the inner membrane by the flippase Wzx encoded by ORF Kr11510, and subsequently polymerized by Wzy encoded by ORF Kr11509. Finally, the expression of the polysaccharide on the cell surface requires the activity of proteins encoded by wza, wzb, wzc and wzi [44]. The names of the enzymes are squared; grey shades correspond to putative enzyme activity. D-Glc: D-glucose ; D-Gal: D-galactose, D-Man: D-mannose, D-GalA: D-galacturonic acid; D-GlcA : D-glucuronic acid ; P-und: undecaprenyl-phosphate.
(0.13 MB TIF)

Table S1 Strains used in this study, and their characteristics.
(0.05 MB XLS)
