# Supplementary figures and images for: PCR-Based Identification of Klebsiella pneumoniae subsp. rhinoscleromatis, the Agent of Rhinoscleroma
Source: PLoS Negl Trop Dis. 2011 May 24;5(5):e1052. doi: 10.1371/journal.pntd.0001052 (PMC3101168; doi:10.1371/journal.pntd.0001052)

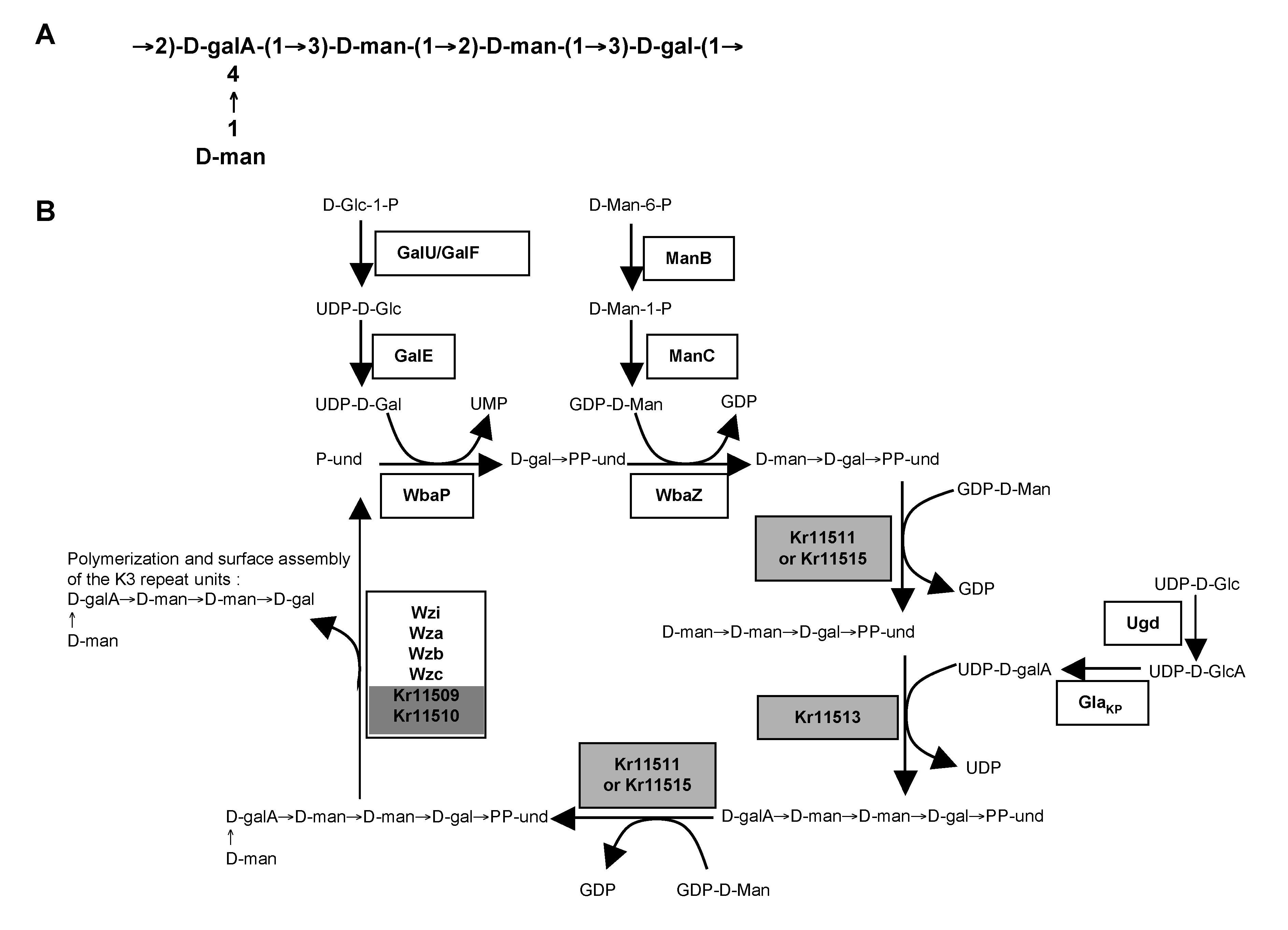

Supplement: Figure S1 — Model of the biosynthesis, polymerization and surface assembly of the K3 capsular polysaccharide. A. Structure of the K3 polysaccharide [42]. B. Possible implication of genes of the cps region in the synthesis and expression of the K3 capsular polysaccharide. In Klebsiella and E. coli [28], [34], the synthesis of UDP-D-galactose from UDP-D-glucose requires protein GalE (UDP-galactose 4-epimerase), with GalF regulating its level [28]. Next, WbaP is involved in the transfer of the D-galactose to undecaprenyl-phosphate, the lipid carrier of repeat units [44]. Synthesis of D-mannose from mannose-6-phosphate is catalyzed by the products of genes manB and manC [44], which are located at the 3′ terminus of the K3 cps region. The (1→3) linkage of the D-mannose to D-galactose is performed by the mannosyl transferase encoded by wbaZ [34]. The linkage of the two additional D-mannose residues is presumably carried out by the putative mannosyl transferases Kr11515 and Kr11511. The remaining putative transferase of the K3 cps cluster with no assigned function, encoded by Kr11513, might be involved in the transfer of UDP-D-galacturonic acid residues on D-mannose. Synthesis of D-galacturonic acid residues is achieved by both Ugd and GlaKP enzymes. The product of gene ugd transforms UDP-D-glucose into UDP-D-glucuronic acid, which can be converted into UDP-D-galacturonic acid through the activity of GlaKP [45]. These capsule unit repeats are then translocated through the inner membrane by the flippase Wzx encoded by ORF Kr11510, and subsequently polymerized by Wzy encoded by ORF Kr11509. Finally, the expression of the polysaccharide on the cell surface requires the activity of proteins encoded by wza, wzb, wzc and wzi [44]. The names of the enzymes are squared; grey shades correspond to putative enzyme activity. D-Glc: D-glucose ; D-Gal: D-galactose, D-Man: D-mannose, D-GalA: D-galacturonic acid; D-GlcA : D-glucuronic acid ; P-und: undecaprenyl-phosphate. (0.13 MB TIF) [file pntd.0001052.s001.tif]
